# Effect of Paprika Xanthophyll Supplementation on Cognitive Improvement in a Multitasking Exercise: A Pilot Study for Middle-Aged and Older Adults

**DOI:** 10.3390/healthcare10010081

**Published:** 2022-01-01

**Authors:** Asako Shirai, Tsuyoshi Wadazumi

**Affiliations:** 1 Department of Sport Education, Osaka University of Health and Sport Sciences, 1-1 Asashirodai, Kumatori, Sennan District, Sennan-gun 590-0496, Osaka, Japan; 2 Faculty of Health and Well-Being, Kansai University, 1-11-1 Kaorigaoka-cho, Sakai-ku, Sakai 590-8515, Osaka, Japan; wadazumi@kansai-u.ac.jp

**Keywords:** supplementation, cognitive function, multitasking exercise, xanthophyll, erythrocyte deformability, oxygen transport efficiency

## Abstract

Ingestion of paprika xanthophyll supplement (PX), which has antioxidant effects, has been recently reported to maintain red blood cell deformability and improve oxygen delivery efficiency. Therefore, we hypothesized that the brain activation induced by multitasking exercise in middle-aged and older participants along with the improved erythrocyte oxygen-carrying efficiency induced by PX supplementation would show a synergistic effect, increasing oxygen supply to the brain and improving cognitive function more effectively. In study 1, cerebral blood flow measurements were conducted during the multitasking exercise and cognitive function tests to verify their effect on cognitive function. The results confirmed that cerebral blood flow increased during the exercise and cognitive function improved after the exercise. In study 2, we compared the effects of the multitasking exercise on cognitive function before and after PX supplementation in middle-aged and older participants to evaluate the effects of PX supplementation. The results suggested that PX supplementation enhanced the effects of active multitasking exercise on cognitive function. We speculate that the improvement of oxygen transport efficiency by PX resulted in more effective oxygen supply, allowing the multitasking exercise to occur more effectively, which was reflected as an improvement in the cognitive function.

## 1. Introduction

Physical activity, especially exercise training such as aerobic exercise, and cognitive activity have been reported to be effective in preventing dementia [1,2,3,4,5]. The increased cerebral blood flow, as a result of continuous exercise, has been reported to improve cognitive function by activating regions involved in the central nervous system functions such as executive function and memory [6,7]. Recent studies have reported that in addition to continuous and aerobic exercise, acute resistance exercise, low- and moderate-intensity exercise [4,5], and multitasking exercise involving simultaneous performance of multiple tasks with exercise can improve cognitive function [7,8].

Nevertheless, the cognitive function under hypoxic conditions such as high-altitude training is significantly lower than that under normoxic conditions because of the limited oxygen supply to the brain [9,10,11,12]. In addition, vascular endothelial dysfunction due to aging has been reported to lead to deterioration of the vessel walls and reduced cerebral blood flow, resulting in a decline in cognitive function [11]. This decline in cognitive function is due to a decrease in oxygen supply due to decreased cerebral circulation [13]. These considerations indicate the importance of suppressing the reduction in cerebral blood flow and maintaining oxygen supply to meet the demands of cerebral metabolism and thereby maintain normal cognitive function [14,15].

Antioxidant supplements have been recently suggested to have anti-aging effects on red blood cells [13,16,17,18]. Carotenoids, in particular, have attracted attention as potent inhibitors of factors that reduce vascular endothelial function [16]. Specifically, xanthophylls may act to maintain the deformability of red blood cells [17,19,20]. Previous studies that examined the effects of xanthophyll ingestion on endurance in athletes reported that the maintenance of erythrocyte deformability because of xanthophyll ingestion may have improved oxygen delivery in athletes and may lead to improvements in endurance exercise performance [21,22,23].

On the basis of these findings, we hypothesized that multitasking exercise with paprika xanthophyll (PX) supplementation in middle-aged and older adults would induce a synergistic effect and increase the oxygen supply to the brain, resulting in effective improvement of cognitive function. Therefore, the present study aimed to (1) confirm the effects of existing multitasking exercises on cognitive function and (2) compare the cognitive effects of multitasking exercises before and after PX supplementation in middle-aged and older adults.

## 2. Materials and Methods

Study 1: To confirm the effects of multitasking exercise on cognitive function.

Study 2: To evaluate the effect of PX supplement ingestion on cognitive improvement through a multitasking exercise.

### 2.1. Purpose

Study 1 aimed to examine the effects of a multitasking exercise (Co-kara exercise: CE) [24] used in a dementia-prevention class in S city on cognitive function.

Study 2 aimed to compare the cognitive effects of CE before and after the 4-week PX supplementation period in middle-aged and older participants.

### 2.2. Study Design

#### 2.2.1. Participants

For study 1, the participants were nine male university students aged 21 years.

For study 2, the participants were 31 middle-aged and older adults living in Osaka Prefecture: six males and 25 females, with a mean age of 62.4 years and an age range of 51 to 74 years (Table 1).

#### 2.2.2. Experimental Design

**Figure 1:**
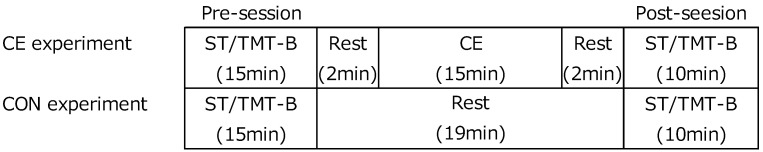
Experimental design (Study1). CE: Co-kara exercise. CON: control, ST: Stroop test, TMT-B: Trail-making test.

In study 1, participants underwent the following two conditions: exercise (CE) and control (CON) (Figure 1). In the CE condition, participants took the Stroop (ST) and Trail-making test (TMT) before (pre-session) and 2 min after (post-session) CE. In the CON condition, participants rested during the interval between pre- and post-sessions. Participants were monitored by Near-infrared Spectroscopy during this experiment. The two conditions were implemented using a crossover design.

**Figure 2:**
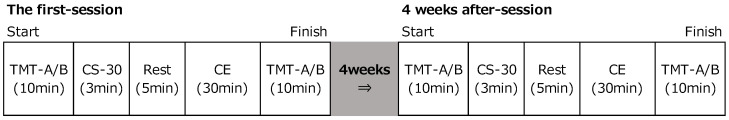
Experimental design (Study 2). CS-30: Exercise task using a chair, CE: Co-kara exercise.

In study 2, to evaluate the effects of an active multitasking exercise with supplementation, two experiments were conducted before and four weeks after supplementation with PX (Figure 2). In the first experiment, participants underwent TMT and performed a chair-rise exercise (CS-30) before CE. Next, CE was carried out for 30 min. After completing the CE, participants underwent TMT again. During the exercise, the heart rate was measured using a wearable activity monitor. In addition, a 16-item questionnaire survey was conducted to evaluate daily life habits, including a survey of medical history, exercise habits, and dietary habits. The same procedure was repeated four weeks later.

- Supplement; A commercial supplement (“OXYDRAIVE”; Glico Nutrition Co., Ltd., Osaka, Japan) containing PX extracted from natural red bell pepper was used in this study. Xanthophyll-containing gelatin capsules (each containing 9 mg of seven types of xanthophylls, including 5 mg of capsanthin and 0.5 mg/g of β-cryptoxanthin), were used in study 2. All participants took the capsules orally every morning with meals for four weeks. PX contains seven xanthophylls (capsanthin, cucurbitaxanthin A, β-cryptoxanthin, zeaxanthin, capsorubin, crypto-capsin, capsanthin, and 3,6-epoxide).
- Multitasking exercise; The multitasking exercise used in this study was “Co-kara exercise: CE, which was devised as a dementia-prevention exercise in S city, Osaka. CE consists of several kinds of basic movements (blocks), such as upper-limb exercises and foot-stomping exercises, which are combined in four beats of two repetitions each, with changes in rhythm and direction. The exercise time (CE) in study 1 was 15 min, while that in study 2 was 30 min.

### 2.3. Measurements

#### 2.3.1. Measurements for Study 1

For study 1, the measurements were as follows:

- Stroop Test; The New Stroop Test II [25], which can collectively measure two indices, ST interference and reverse ST interference, was used to assess cognitive function. This test involves the following two tasks: (1) identification of the color represented by a word and the color in which the letters are written and (2) identification of options showing inconsistency between the color of the letters and the meaning implied by the word (ST interference). The ability to handle this ST interference is calculated as the difference in performance between the incongruent and congruent tasks and is evaluated as an executive function. In study 1, we compared the ST interference rate with the reverse ST interference rate.
- Trail-making test; The TMT was used to assess cognitive function [26]. The TMT consists of the following two parts: Part A (TMT-A) consists of numbers only, in the order of “1–25,” while Part B (TMT-B) in the Japanese version consists of numbers and Hiragana in Japanese, in the order of “1-A-2-I-3-U....”. The time to complete each part of the TMT was recorded, and raw time scores were used as the dependent variables. The TMT is a well-established neuropsychological assessment method that can comprehensively evaluate attention, working memory, spatial exploration, and processing speed. In particular, the TMT-B can be used to characterize attentional conversion ability and executive function. The TMT-B has been used in many studies [27,28,29]. In study 1, only the TMT-B was used; practice sessions were conducted to avoid the influence of learning effects in the TMT.
- Near-infrared Spectroscopy; Changes in oxygenated, deoxygenated, and total hemoglobin in blood in the frontal region of the brain during each trial were detected using the double channel of a near-infrared oxygenation monitor (NIRO-200; Hamamatsu Photonics KK, Hamamatsu, Japan). The NIRO probe was fixed to both sides of the forehead with double-sided tape and then firmly fixed with an elastic bandage to prevent it from floating. In this study, the mean value per minute of the change in total hemoglobin, which is the sum of the measurements of oxygenated and deoxygenated hemoglobin obtained from NIRO, was used as an index.

#### 2.3.2. Measurements for Study 2

For study 2, the measurements taken were as follows:

- TMT-A and TMT-B; Cognitive function tests (TMT-A, B) were conducted the following four times: before the first CE (before/pre), after CE (before/post), and before and after CE conducted four weeks later (four weeks/pre and four weeks/post, respectively). As in study 1, sufficient practice sessions were conducted to avoid the influence of learning effects in TMT. The TMT procedure was conducted according to the Japanese version of the manual [27] as follows: the participants were administered the practice questions for part A; after they completely understood part A, they were asked to attempt the questions from the actual part A. After completing the questions from part A, they were administered the practice questions for part B, and after they had understood part B, they were asked to attempt the questions from the actual part B, which was the same version as part A. After the CE, another version of the TMT was conducted in the same order (part A followed by part B). In this assessment, the practice questions were not administered. After four weeks of PX supplementation, the entire TMT procedure, including the order of the tests and the use of practice questions only for the pre-CE assessments, was repeated with two additional versions of the TMT.
- Standard questionnaire survey; The questionnaire consisted of 16 items (Table 2), and it evaluated medical history and smoking habits, exercise habits, and dietary habits among the items listed in the specific health checkup specified in the standards for the implementation of specific health checkups and specific health guidance (2007, Ministry of Health, Labour and Welfare, Ordinance No. 157) [30]. Questionnaires were distributed at the first survey and collected four weeks later. Responses were based on a two-response model (yes/no).
- CS-30; The CS-30 was determined as an index of lower limb muscle strength and exercise tolerance. The CS-30 has been shown to indicate exercise tolerance in patients with chronic respiratory diseases, as well as an index of lower limb muscle strength, and can be used to estimate the amount of physical activity by the number of steps commensurate with the exercise tolerance [31]. CS-30 evaluations were performed before and once after four weeks of PX supplementation.
- Heart rate; Heart rate measurement data were obtained using a wearable activity monitor equipped with an optical heart rate sensor (Huawei BAND 4: Huawei Technologies Japan K.K., Tokyo, Japan). The heart rate during the experiment, at rest, after CS-30 evaluation, during CE, and after CE was measured using the wearable activity monitor.

### 2.4. Statistical Analysis

In study 1, for the comparison of cerebral blood flow, paired t-tests were performed for the mean values per minute at rest and during CE. For comparison of TMT and ST, paired t-tests were performed for the mean values between the pre-and post-session in each trial. SPSS Statistics 27 (IBM Corporation) was used for statistical analyses. The level of statistical significance was set at a *p* < 0.05.

In study 2, for the comparison of the cognitive function before and after the multitasking exercise, the mean TMT-A and TMT-B scores pre-and post-CE exercise were compared using a paired t-test (before/pre vs. before/post). The mean TMT-A and B scores at four weeks of PX supplementation were also compared similarly (four weeks/pre vs. four weeks/post). A two-way repeated measures analysis of variance was used to examine the effect of PX supplementation on heart rate (heart rate vs. time). A Greenhouse–Geisser test was also performed for equality of variance of the within-subject factors. When a significant interaction or effect was observed, multiple comparisons were performed using the Bonferroni correction. The baseline TMT score was used to determine the rate of change in the TMT score after four weeks; for items with a high correlation rate, an independent t-test was performed by the grouping of lifestyle. SPSS Statistics 27 (IBM Corporation) was used for performing these statistical analyses. The level of statistical significance was determined using a two-tailed test with a risk rate of *p* < 0.05. 

### 2.5. Ethics

This study was conducted in compliance with the tenets of the Declaration of Helsinki on the basis of ethics, human rights, and the protection of the personal information of participants. Ethical approval for this study was obtained from the Ethics Committee of the Kansai University Faculty of Health and Well-being (Approval No. 2019-01). 

## 3. Result

### 3.1. Results of Study 1

**Figure 3:**
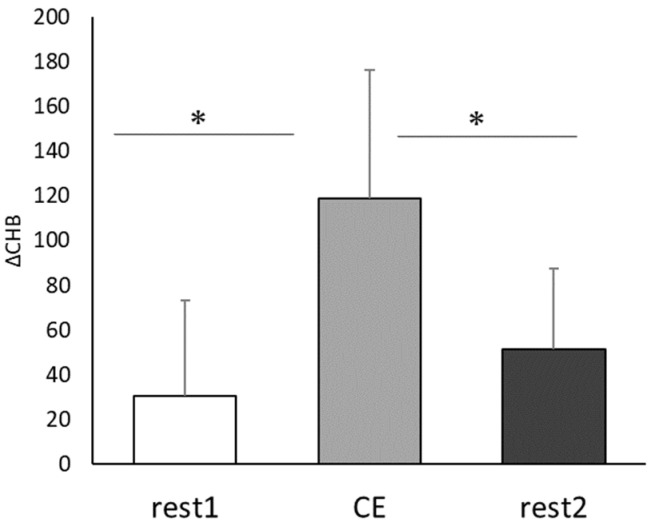
Comparison of changes in cerebral blood flow caused by Co-kara exercise. ΔCHB: the mean value per minute of the change in total hemoglobin; CE: 15 min Co-kara exercise; rest 1:2 min rest before CE; rest 2:2 min rest after CE. (*n* = 9) rest1 vs. CE, * *p* < 0.05 (*p* = 0.012, r = 0.74), rest 2 vs. CE * *p* < 0.05 (*p* = 0.03, r = 0.66).

Based on the Near-infrared Spectroscopy results, the mean cerebral blood flow was 30.7 ± 44.9 at rest before CE, 118.7 ± 60.1 during CE, and 51.7 ± 37 at rest after CE (Figure 3). Thus, the mean cerebral blood flow during CE was significantly higher than that in the Con condition (t(8) = 3.12, *p* < 0.05, r = 0.74), and it significantly decreased after CE (t(8) = 2.51, *p* < 0.05, r = 0.66). 

**Figure 4:**
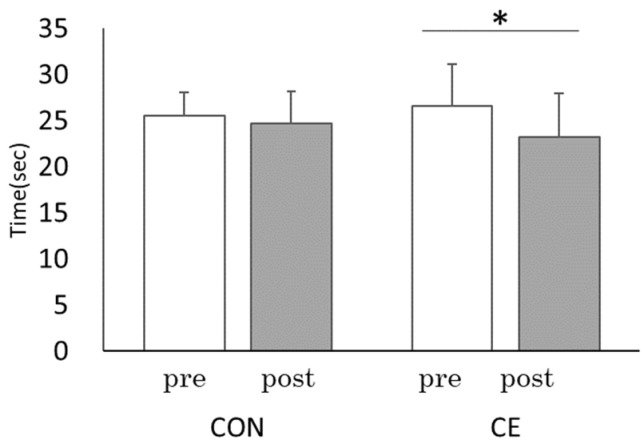
Comparisons of each trial between pre and post: TMT-B. CE: Co-kara exercise experiment; CON: Control experiment; pre: before Co-kara exercise; post: after Co-kara exercise. (*n* = 9) pre- vs. post, CE: * *p* < 0.05 (*p* = 0.018, r = 0.73), CON: not significant (*p* = 0.38, r = 0.31).

**Figure 5:**
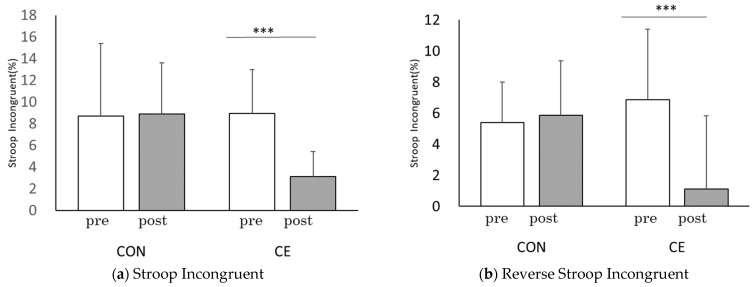
Comparisons of each trial between pre and post Co-kara exercise: Stroop test; (**a**) ST interference rate, (**b**) Reverse-ST interference rate. CE: Co-kara exercise experiment; CON: Control experiment; pre: before Co-kara exercise; post: after Co-kara exercise. (*n* = 9), *** *p* < 0.001. (**a**) Significant difference between pre and post, CE; (*p* = 0.000, r = 0.92), CON; n.s. (not significant, *p* = 0.88, r = 0.19). (**b**) Significant difference between pre and post, CE; (*p* = 0.021, r = 0.71), CON; n.s. (not significant, *p* = 0.723, r = 0.13).

In the pre-and post-CE results, TMT-B mean score performance improved significantly from pre-CE (mean [M] = 26.57, standard deviation [SD] = 4.39) to post-CE (M = 23.24, SD = 2.63; t(8) = 2.98, *p* < 0.05, r = 0.73) (Figure 4). Similarly, the pre- and post-CE comparison of the ST interference rate showed a significant improvement from pre-CE (M = 8.96, SD = 4.26) to post-CE (M = 3.12, SD = 2.45; t(8) = 6.52, *p* < 0.001, r = 0.92). The reverse ST interference rate also significantly improved from pre-CE (M = 6.86, SD = 4.83) to post-CE (M = 1.13, SD = 4.97; t(8) = 2.87, *p* < 0.05, r = 0.71) (Figure 5). Under the CON condition, there was no significant improvement in the TMT score: pre- (M = 25.48, SD = 1.91), post- (M = 24.65, SD =2.55); t(8) = 0.98, *p* = 0.38, r = 0.31, ST interference rates; pre- (M = 8.69, SD = 7.08), post- (M = 8.89, SD = 4.99; t(8) = −0.16, *p* = 0.88, r = 0.19), and the reverse ST interference rate; pre- (M = 5.40, SD = 2.74) and post- (M = 5.85, SD = 3.73; t(8) = −0.367, *p* = 0.72, r = 0.13). Thus, the cerebral blood flow increased during CE, and cognitive function improved after CE.

### 3.2. Results of Study 2

#### 3.2.1. Attributes of the Participants

There were 31 participants (six males and 25 females), with a mean age of 62.4 years and an age range of 51 to 74 years. Eight participants were in their 50s, 26%, 19 participants were in their 60s, 61%, and 13% were in their 70s (Table 1).

#### 3.2.2. Cognitive Task

**Figure 6:**
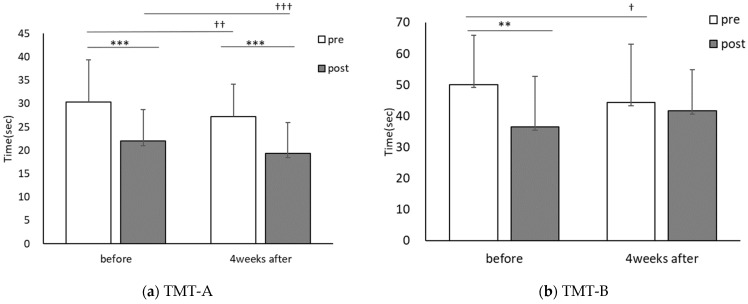
Comparisons of each trial before and the 4-week PX supplementation period: TMT; pre: before CE; post: after CE, before: the first session; 4 weeks after: after the 4-week PX supplementation period. (*n* = 31) before vs. 4-weeks after, † *p* < 0.05, †† *p* < 0.01, ††† *p* < 0.001, pre- vs. post, ** *p* < 0.01, *** *p* < 0.001. (**a**) Significant difference between pre and post, before; (*p* = 0.000, r = 0.84), four weeks after; (*p* = 0.000, r = 0.91), significant difference before and four weeks after, pre; (*p* = 0.009, r = 0.46), post; (*p* = 0.000, r = 0.58). (**b**) Significant difference between pre and post, before; (*p* = 0.006, r = 0.47), four weeks after; (*p* = 0.229, r = 0.22), significant difference before and four weeks after, pre; (*p* = 0.039, r = 0.37), post; (*p* = 0.061, r = 0.33).

The TMT-A score was 30.4 ± 9.1 for pre-CE and 22.0 ± 6.8 for post-CE in the first session and 27.2 ± 7.1 for pre-CE and 19.3 ± 6.6 for post-CE in the session conducted four weeks later. CE intervention significantly improved the scores in both the first session (t(30) = 8.651, *p* < 0.001, r = 0.84) and the session conducted four weeks later (t(30) = 12.228, *p* < 0.001, r = 0.91). Comparisons of the TMT-A scores before and after PX supplementation showed significant improvement for both pre-CE (t(30) = 2.816, *p* < 0.01, r = 0.46) and post-CE (t(30) = 3.943, *p* < 0.001, r = 0.58). The TMT-B score was 50.1 ± 16.1 for pre-CE and 42.2 ± 16.7 for post-CE in the first session and 45.2 ± 16.7 for pre-CE and 41.7 ± 13.4 for post-CE in the session conducted four weeks later. TMT-B scores improved significantly in the first session by the CE intervention (t(30) = 2.931, *p* < 0.01, r = 0.47) and with no significant difference in the session conducted four weeks later (t(30) = 1.227, *p* = 0.23, r = 0.22). Comparison of TMT-B scores pre-and post-PX supplementation showed that the pre-CE (t(30) = 2.157, *p* < 0.05, r = 0.37) and post-CE (t(30) = 1.946, *p* = 0.06, r = 0.33) significantly improved before the first session of CE (Figure 6).

#### 3.2.3. CS-30 and Heart Rate

**Figure 7:**
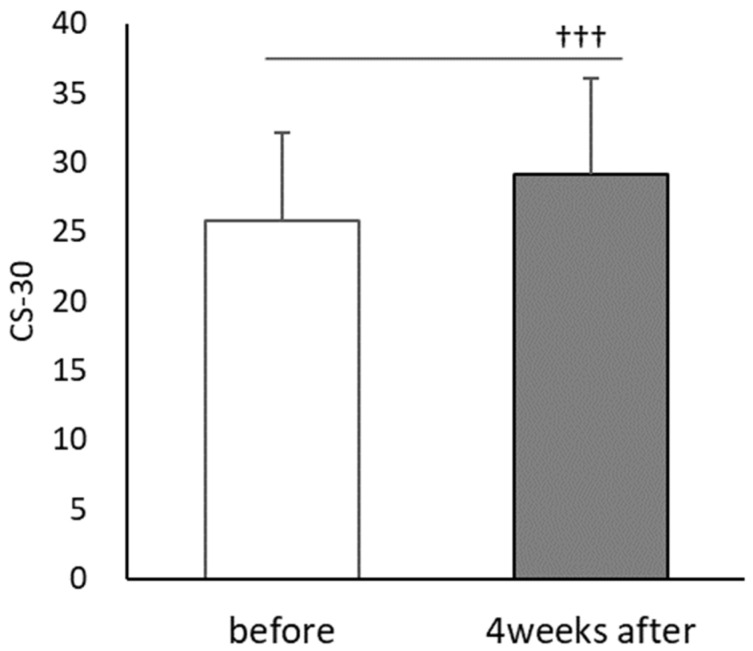
Comparisons of each trial in CS-30 before and after the 4-week PX supplementation period; before: the first session; 4 weeks after: after the 4-week PX supplementation period. (*n* = 31); before vs. 4 weeks after: ††† *p* < 0.001 (*p* = 0.000, d = 0.73).

**Figure 8:**
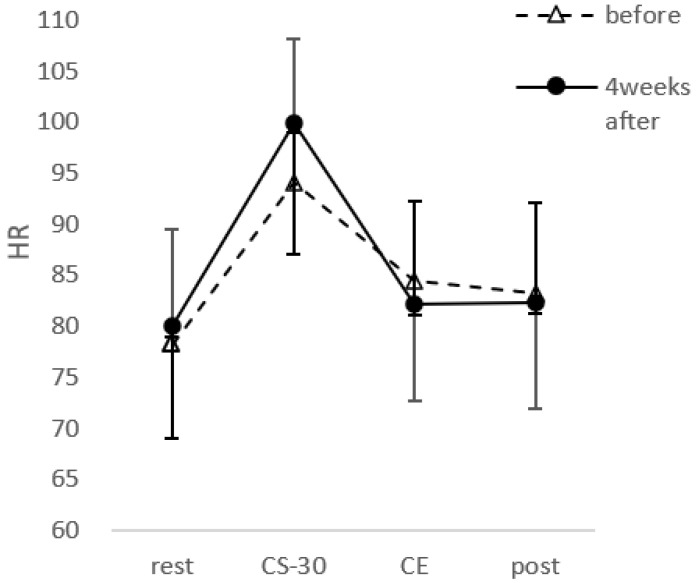
Heart rate across each point for before and after the 4-week PX supplementation period. Data were analyzed using two-way ANOVA. HR: heart rate, rest: before exercise, CS-30; immediately after CS-30 exercise, CE: during Co-kara exercise, post; after Co-kara exercise. CE was measured using the wearable activity monitor. There was a main effect of time (*p* < 0.01) and an interaction (*p* < 0.01).

CS-30 significantly improved after PX supplementation (t(30) = 5.922, *p* < 0.001, d = 0.73) with a score of 25.8 ± 6.4 during the first session and 29.19 ± 7.0 after PX supplementation (Figure 7). Heart rate showed a main effect of time (F(1,23) = 64.882; *p* < 0.01, $\eta_{p}^{2}$ = 0.691), but no main effect of PX supplementation (F(1,23) = 0.039; n,s,$\eta_{p}^{2}$ = 0.001). There was also a significant interaction between PX supplementation and time (F(1,23) = 5.548; *p* < 0.01, $\eta_{p}^{2}$ = 0.161) (Figure 8).

#### 3.2.4. Changes in the TMT Score before and after PX Supplementation

**Figure 9:**
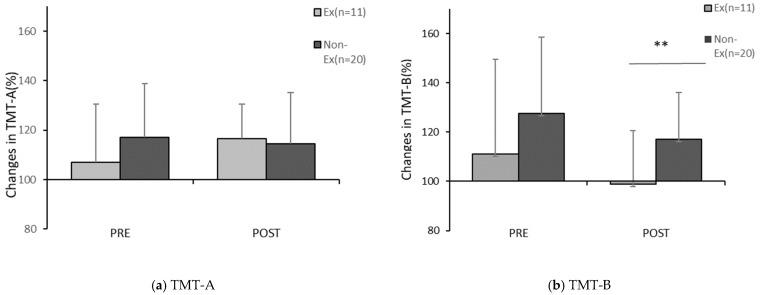
Comparison of changes in the TMT score before and after PX supplementation. Ex: the habitual exerciser group; NonEx: non-habitual exerciser group. Comparison before and four weeks after PX supplementation, (**a**) Changes in TMT-A after PX supplementation, (**b**) Changes in TMT-B after PX supplementation in each trial; Ex vs. NonEx: ** *p* < 0.05. (**a**) Significant difference between Ex and Non-Ex, pre; n.s. (*p* = 0.25, r = 0.21), post; n.s. (*p* = 0.76, r = 0.), (**b**) Significant difference between Ex and Non-Ex; pre; n.s. (*p* = 0.21, r = 0.23), post: (*p* = 0.02, r = 0.41).

The correlation between lifestyle factors and the changes in the TMT scores before and after PX supplementation are shown in Table 2. Significant correlations were found for the following two items: “Exercise habit of lightly sweating for more than 30 min at least two days a week for more than one year (Exercise habit)” (r = −0.455, *p* < 0.05) and “Skip breakfast” (r = 0.362, *p* < 0.05). Comparison between groups by a t-test for each of these factors showed significant differences in relation to the presence of exercise habit and the rate of change in the post-exercise TMT-B score after four weeks (*p* < 0.05) and no significant difference in relation to the Skip breakfast component in the pre-exercise TMT-A (n.s.) (Table 2, Figure 9).

## 4. Discussion

In study 1, we examined the effects of an existing multitasking exercise (CE) on cognitive function, and in study 2, we compared the effects of CE on cognitive function before and after PX supplementation in middle-aged and older adults to clarify the effects of PX supplementation. The results confirmed the effect of CE on cognitive function and indicated a significant performance improvement in the cognitive tasks performed after PX supplementation.

### 4.1. Synergistic Effects of Multitasking Exercise and PX

Active multitasking exercise has been reported to activate the frontal lobe of the brain and enhance executive functions, and exercise combined with music has been reported to be more effective than exercise alone in improving cognitive function [7]. In study 1, cerebral blood flow during CE was significantly higher than that at rest (Figure 3), and the TMT and ST scores significantly improved after CE (Figure 4 and Figure 5). These results were similar to those obtained in a previous study [32], and the improvement effect of CE on cognitive function was confirmed. 

In study 2, TMT-A and B before CE were significantly improved after four weeks compared to before PX supplementation, and similarly, TMT-A was improved after CE (Figure 6). CS-30 significantly improved after four weeks (Figure 7). 

### 4.2. Effect of PX Supplementation

The research of “nutritional cognitive neuroscience”, a new field of interdisciplinary research on the effects of many aspects of nutrients on the structure and function of the brain, has recently shown remarkable progress [33,34]. Previous studies in this field have reported various aspects of the effects of carotenoids on cognitive functions [35,36]. Most of these studies suggested that the “neuroprotective mechanism” of carotenoids, which is mediated by their antioxidant and anti-inflammatory effects, influences the improvement in cognitive function, and it is being investigated that carotenoid nutrients may directly contribute to the protection of brain neurons. While most of the carotenoids used in previous studies were consumed alone or in combination (e.g., lutein zeaxanthin, and astaxanthin), in this study, seven types of carotenoids (capsanthin, cucurbitaxanthin A, β-cryptoxanthin, zeaxanthin, capsorubin, crypto-capsin, capsanthin, and 3,6-epoxide) were examined. A previous study that examined the effects of PX administered to endurance athletes for one month showed that heart rate during 30 min of steady-state exercise (60% VO_2_ peak) decreased after PX supplementation, and improvements in carrying efficiency and overall endurance performance have been reported [21,22,23]. We speculate that one of the factors underlying this effect of PX was the maintenance of erythrocyte deformability and the resultant increase in oxygen transport efficiency and exercise performance. The evidence for the maintenance of erythrocyte deformability is based on findings showing that PX supplementation increases the localization of multiple xanthophylls in erythrocyte oil membranes [22,37], which may reduce erythrocyte peroxidation and maintain erythrocyte deformability [13,38]. These results suggest that maintenance of erythrocyte deformability, improvement of oxygen transport efficiency, and an increase in oxygen supply to the brain due to the antioxidant effect of PX may have indirectly contributed to the improvement of cognitive function in the present study.

One of the factors through which improvements in erythrocyte deformability improve oxygen delivery efficiency is an improvement in the ability to transport blood through the capillaries. When erythrocyte deformability is reduced due to the oxidation of erythrocyte oil films, erythrocytes cannot pass through small capillaries [13]. Antioxidant effects have been suggested to facilitate erythrocyte deformability and allow oxygen transport throughout the body in capillaries, resulting in improved exercise efficiency and overall endurance [21]. It is likely that these mechanisms were also reflected in the results of study 2.

While cerebral blood flow tends to decrease with age, PX supplementation was shown to maintain erythrocyte deformability and improve oxygen supply to the brain, although it did not change cerebral blood flow. Furthermore, brain activity was enhanced after PX supplementation, which may have led to an improvement in cognitive function through the synergistic effect of PX supplementation and CE.

We focused on the relationship between lifestyle and changes in TMT scores to examine the effects of PX supplementation and CE on cognitive function (Table 2). The significant difference in the rate of change in the cognitive task (TMT-B) score after PX supplementation, in relation to the presence or absence of the exercise habit, confirmed that cognitive task performance tended to improve after PX supplementation in participants who exercised less, in addition to suggesting that the antioxidant effects of PX affect cognitive function. These findings could have been noted because participants who exercised less showed a decline in vascular endothelial function due to aging [39] and may have been affected more by PX supplementation. Furthermore, the difference between the results of TMT-B and TMT-A may be explained by previous studies [40], which observed that the TMT-B task is involved in the executive function and that TMT-B has increased activation of cerebral blood flow as compared to TMT-A. On the other hand, the significant difference in the rate of change in cognitive task performance (TMT-A) after PX supplementation, in relation to the item “whether or not breakfast is consumed,” may be related to the nutrients in PX. Detailed data on participants’ dietary habits and amount of physical activity are required to clarify the mechanisms underlying these findings, which will be the subject of further research.

The present study examined the effects of PX supplementation on cognitive function in healthy middle-aged and older adults. The findings of the present study suggested that a combination of multitasking exercise and PX supplementation could maintain and improve cognitive function.

### 4.3. Limitations of the Study

From 2020 to 2021, the coronavirus pandemic made participant recruitment and study continuation quite difficult, and we were unable to conduct a placebo experiment as a result. In addition, the subjects were middle-aged and older Japanese adults, and there was a sex bias. Moreover, we could not measure the xanthophyll distribution in the blood and cerebral blood flow and the deformability of red blood cells when xanthophylls were ingested. These aspects need to be clarified in future studies.

## 5. Conclusions

Active multitasking exercise and PX supplementation may have positive effects on cognitive function. The synergistic effect of multitasking exercise and PX supplementation on brain activity and cognitive function may be attributable to the enhancement of oxygen delivery efficiency by maintaining the deformability of red blood cells in the blood.

## Figures and Tables

**Table 1:** Physical characteristics of the participants (SD—standard deviation).

Subject	Sex	Age (Year) Mean; Age Range	Hight (cm) Mean ± SD	Weight (kg) Mean ± SD
31	All	62.4; 51–74	160.0 ± 7.7	57.9 ± 11.8
6	Male	63.7; 52–73	170.0 ± 4.9	73.6 ± 9.8
25	Female	62.0; 51–74	158.0 ± 6.1	54.1 ± 8.65

**Table 2:** Correlation between lifestyle and changes in the TMT score.

Questions			Changes in the TMT Score before and after PX Supplementation			
	Number		Before		Four Weeks after	
	No	Yes	TMT-A	TMT-B	TMT-A	TMT-B
Are you currently taking a drug to lower blood pressure?	21	10	−0.351	−0.214	−0.184	0.176
Are you currently taking a drug to lower blood glucose or insulin injections?	30	1	0.084	0.150	0.121	0.105
Are you currently taking a drug to lower cholesterol or neutral fat?	26	5	−0.318	0.144	0.195	0.036
Have you ever been told by a doctor that you have a stroke (e.g., cerebral hemorrhage, cerebral infarction) or have you ever received treatment for a stroke?	31	0	-	-	-	-
Have you ever been told by a doctor that you have heart disease (e.g., angina, myocardial infarction) or have you ever received treatment for heart disease?	30	1	0.104	0.073	−0.204	−0.001
Have you ever been told by a doctor that you have chronic renal failure/renal insufficiency or have you ever received treatment for chronic renal failure (dialysis, etc.)?	31	0	-	-	-	-
Have you ever been told by a doctor that you have anemia?	28	3	0.222	0.067	0.185	0.156
Are you a current regular smoker?	31	0	-	-	-	-
Have you gained ≥10 kg since you were 20 years old?	18	13	0.055	0.213	−0.121	−0.042
Have you been exercising at least two days per week, at least 30 min each at an intensity that causes a slight sweat, for at least one year?	20	11	−0.202	−0.333	0.106	−0.455 *
Do you walk for at least 1 h every day or have equivalent physical activities in your daily life?	19	12	−0.088	−0.013	0.128	−0.108
Do you walk faster than people of your age and sex?	15	16	0.083	−0.166	0.035	0.022
Do you have an evening meal within 2 h before bedtime three days or more per week?	23	8	0.332	0.325	0.087	0.216
Do you have any snacks or sweet beverages other than breakfast, lunch, and dinner?	15	13	0.298	−0.273	−0.114	0.088
Do you skip breakfast three days or more per week?	24	7	0.362 *	0.220	0.125	−0.323
Do you feel refreshed after a night’s sleep?	11	19	0.127	−0.171	0.098	−0.072

*. Correlation is significant at the 0.05 level.

## Data Availability

The data presented in this study are available on request from the corresponding author and the permission of all parties involved in the study.
